# Iron-Chelated Polydopamine Decorated Doxorubicin-Loaded Nanodevices for Reactive Oxygen Species Enhanced Cancer Combination Therapy

**DOI:** 10.3389/fphar.2019.00075

**Published:** 2019-02-06

**Authors:** Xu-Jing Li, Wen-Tong Li, Zi-Hao-Ran Li, Li-Ping Zhang, Cheng-Cheng Gai, Wei-Fen Zhang, De-Jun Ding

**Affiliations:** ^1^Department of Pathology, Weifang Medical University, Weifang, China; ^2^Collaborative Innovation Center for Target Drug Delivery System, Weifang Medical University, Weifang, China; ^3^College of Pharmacy, Weifang Medical University, Weifang, China

**Keywords:** polydopamine, combination therapy, reactive oxygen species, doxorubicin, breast cancer

## Abstract

Combination therapy which enhances efficacy and reduces toxicity, has been increasingly applied as a promising strategy for cancer therapy. Here, a reactive oxygen species (ROS) that enhanced combination chemotherapy nanodevices was fabricated based on the Fe-chelated polydopamine (PDA) nanoparticles (NPs). The structure was characterized by dynamic light scattering-autosizer, transmission electron microscopy, energy dispersive spectroscopy, and Fourier-transform infrared (FT-IR) spectrophotometer. The *in vitro* drug release profile triggered by low intracellular pH indicated that the system demonstrated controlled therapeutic activity. *In vitro* cell uptake studies showed that doxorubicin (DOX)-loaded Fe-PDA/ folic acid (FA)- polyethylene glycol (DOX@Fe-PDA/FA-PEG) had a strong uptake capacity and can be rapidly internalized by MCF-7 cells. The *in vitro* experiments demonstrated that DOX@Fe-PDA/FA-PEG triggered the intracellular ROS overproduction, thereby enhancing its therapeutic effect on breast cancer. In summary, this experiment demonstrated the novel DOX-loaded composite NPs used as a potential targeted nanocarrier for breast cancer treatment, which could be a promising therapeutic strategy against breast cancer.

## Introduction

As one of the most common malignant tumors among women, breast cancer is the second and common cause of cancer-related death in women (Wood et al., 2017; Bray et al., 2018). Chemotherapy has become one of the most mature and common treatment option for breast cancer (Fisher et al., 1998; Miller et al., 2016; Spiegel and Koontz, 2018). Doxorubicin (DOX) is an anthracycline non-specific broad-spectrum anticancer drug that is widely used to treat breast cancer. Doxorubicin can exert its effects by elevating reactive oxygen species (ROS) thereby activating of caspase and ultimately leading to apoptosis (Russell and Cotter, 2015; Chakravarti et al., 2016). However, serious side effects, such as myelosuppression, cardiotoxicity, and drug resistance, are the major clinical chemotherapeutic drawbacks of DOX.

It has been proposed that combination therapeutics plays a synergistic effect and can enhance efficacy and reduce the toxicity of chemotherapy (Xu et al., 2015; Camacho et al., 2016; Kemp et al., 2016; Seo et al., 2017). Dayton et al. (2011) reported that the use of HO-3867, which is a synthetic curcumin analog, combined with DOX, in low doses to achieve enhanced cell death and reduced myocardial toxicity. And the increased generation of ROS, thereby resulting in oxidative damage to the cellular constituents, is widely exploited for therapeutic benefits on cancer (Matés and Sánchez-Jiménez, 2000; Schumacker Paul, 2015; Zhou et al., 2016). Fe, which plays a role in several types of cell death, has long been associated with toxicity because it induces hydroxyl radical (OH⋅), which is a ROS formed via Fenton reaction (Dixon and Stockwell, 2013; Shen et al., 2018; Zhang et al., 2018). Using ROS-producing agents could enhance the anticancer activity of DOX in cancer therapy through ROS-mediated apoptosis (Xia et al., 2017; Wu et al., 2017), autophagy (Fong et al., 2012), and ferroptosis (Zheng et al., 2017). Fan et al. (2014) identified the synergistic effect of DOX/ selenocystine sensitized to DOX by through ROS overproduction. Dai et al. (2018) fabricated assembled metal-phenolic network Nps as a novel ROS promoted synergistic nanomedicine platform for cancer therapy. This observation inspires us to import an iron-supply system in combination with DOX to elicit a synergistic effect on the cancer therapy.

Recently, researchers attempted to build some drug carrier systems to load and transport DOX overcoming the low bioavailability, poor absorption, and high toxicity of DOX (Xu et al., 2015; Kemp et al., 2016; Indermun et al., 2018). Particularly, polydopamine (PDA), which is a natural-inspired polymer, is an appealing material as drug carrier due to its good biocompatibility (Lynge et al., 2015; Indermun et al., 2018; Ryu et al., 2018). Considering its abundant aromatic rings, PDA NPs could be an efficient platform for loading DOX through π–π stacking and hydrogen-bonding interactiron. Meanwhile, the existence of phenolic hydroxyl groups on the surface makes it suitable for further modification with PEG, which could endow nanoparticles excellent physiological stability of NPs (Liu et al., 2014). More attractively, the phenolic surface have excellent chelating ability with metal ions such as Mn (Miao et al., 2015; Xi et al., 2017), Cu (Ge et al., 2017), and Fe (Li et al., 2016).

**FIGURE 1:**
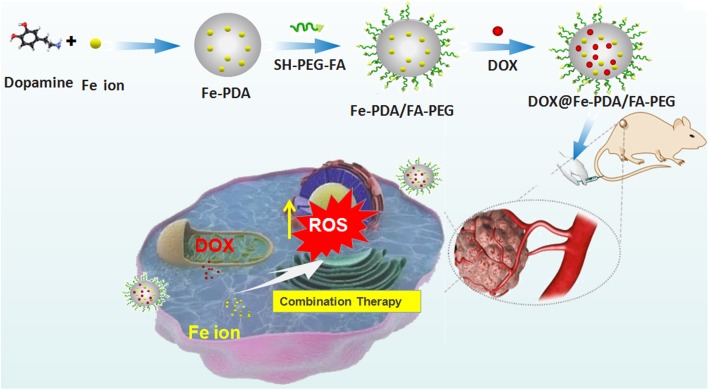
Schematic representation of DOX@Fe-PDA/PEG-FA synthesis, targeted cell uptake and intracellular drug release and combination therapy.

Keep all the issues in mind, we hypothesized that the Fe-chelated PDA nanoparticles with DOX loading could act as an Fe-supply system used for Fe and DOX combined cancer theranostics, as shown in Figure 1. The designed DOX@Fe-PDA/folic acid (FA)-PEG could be provided with several advantages, as follows: (Wood et al., 2017) Combination therapy. The chemotherapy drug DOX undergoes redox cycles to generate and increase H_2_O_2_ in living cells. The released Fe from PDA further reacts with H_2_O_2_ to generate hydroxyl radical via Fenton reaction and induces cell death. In combination with Fe, DOX was prone to kill cancer cells efficiently (Bray et al., 2018). Biocompatibility and safety. PDA, which is a natural biopolymer, possesses biocompatibility. The coated PEG and chelated Fe of PDA Nps were metabolic. Meanwhile, the pH-triggered release performance of PDA in tumor microenvironment, avoids damage to surrounding tissues. The PEG-coating can help Nps to ameliorate long-term circulation (Fisher et al., 1998). Tumor targeted. Considering folate receptor overexpression on the surface of breast cancer cells, the FA conjugated NPs may improve cell uptake via receptor mediated endocytosis. In summary, the DOX@Fe-PDA/FA-PEG system could be used as potential combination chemotherapy nanodevice for breast cancer treatment.

## Materials and Methods

### NPs Synthesis

The synthesis of NPs was modified based on the previously introduced procedure (Li et al., 2016). In brief, 4.08 mg FeCl_3_ and 15 mg dopamine plus 10 mL of water were mixed and stirred at room temperature for 1 h. Then 500 mg Tris was added, and the mixture was stirred at room temperature for 1.5 h. The mixture was centrifuged at 12000 rpm for 15 min to obtain Fe-PDA NPs. A total of 3.85 mL Fe-PDA NPs (5.2 mg/mL) were mixed with 20 mg FA-PEG-SH, 4.7 mg Tris, and 100 μL *tris*(2-carboxyethyl)phosphine (8 mg/mL). The mixture was vigorously stirred for 1 h at room temperature. Then, the FA-PEG modified NPs (Fe-PDA/PEG-FA) were purified via centrifugation and washed with deionized water.

### Drug Loading

A total of 2 mg adriamycin hydrochloride were added into 300 μL of dimethyl sulfoxide and 8.2 μL of triethylamine was added. The mixture was stirred in dark at room temperature for 12 h to desalinate hydrochloride. Then, the neutral DOX (2 mg) above-mentioned was added dropwise to 1 mL of Fe-PDA/FA-PEG NPs (10 mg/mL). Afterward, Tris (2.42 mg) was added and volume of 3 mL was obtained by adding distilled water. After vigorous stirring for 24 h in the dark, free DOX was removed via centrifugation at 12000 rpm for 10 min, then washed with phosphate buffer solution (PBS) and stored at 4°C in the dark. The DOX loading capacity of NPs was determined by UV-Vis spectrophotometer at the wavelength of 480 nm. The encapsulation efficiency (EE) of DOX was calculated by the following equation: EE = (initial amount of feeding drugs – free drugs)/initial amount of feeding drugs.

### NPs Characterization

The size and Zeta potential of the prepared NPs were measured by dynamic light scattering-autosizer (DLS) on Zetasizer Nano ZS90 (Malvern Instruments, Malvern, United Kingdom). The liquid sample was sonicated before measurement. Three independent test results were recorded. The shape and surface morphology of the NPs were imaged by a transmission electron microscope (TEM, JEM-1230; JEOL, Tokyo, Japan). TEM, energy dispersive X-ray spectroscopy (EDS) and corresponding EDS-mapping were adopted for morphology and elemental distribution analyses on the JEM-1230 electron microscope operated at 200 kV. The chemical composition and structural changes of NPs were analyzed by Fourier transform infrared (FT-IR) spectroscopy (VERTEX 70; Bruker, Bremen, Germany). The IR spectra of the samples were obtained in the range of 4000 and 500 cm^-1^.

### *In vitro* Drug Release Profiles

The *in vitro* DOX release behavior of DOX@Fe-PDA/FA-PEG was tested as reported previously (Liu et al., 2014). Briefly, DOX@Fe-PDA/FA-PEG was dispersed in 2 mL PBS with the pH of either 7.2 or 5.5. The tube was shaken at 37°C with 100 rpm in dark. At appropriate time points, the full release buffer was collected via centrifugation at 12000 rpm for 10 min, and replaced with 2 mL of fresh PBS. The amount of released drug DOX was quantified by a UV spectrophotometer at the wavelength of 480 nm. The correlation between the accumulative DOX released from NPs and time was plotted.

### Cell Culture

The *in vitro* cell cytotoxicity cellular uptake and ROS measurement were assessed on human breast cancer cell line MCF-7, which was purchased from American Type Culture Collection. Cells were incubated at 37°C with modified Eagle’s medium (MEM) containing 10% fetal bovine serum (FBS), 100 U/mL penicillin, and 100 mg/mL streptomycin in a 5% CO_2_ atmosphere.

### Cellular Uptake Study

A total of 2 × 10^5^ cells/well MCF-7 cells were seeded in 6-well plates for 24 h. Then, the samples (free DOX, DOX@Fe-PDA/FA-PEG) were added to each well (equivalent DOX concentration of 10 μg/mL) and the cells were incubated at 37°C for an appropriate time at an additional of 24 h. Afterward, the cells were washed with PBS and stained by Hoechst 33342 (Sangon Biotech, Shanghai, China). Confocal laser scanning microscopy (CLSM) imaging was performed on LSM 410 fluorescence microscope (Zeiss, Jena, Germany). The fluorescence signal of DOX was excited at 488 nm and measured at 610 nm. The fluorescence signal stained by Hoechst 33342 was excited at 405 nm and detected at 490 nm.

### *In vitro* Cytotoxicity by Using MTT Assay

MCF-7 cells were seeded in 96-well plates at a density of 5000 cells per well and incubated in 100 mL of medium for 24 h to allow attachment. Then, the cells were incubated with free DOX and DOX@Fe-PDA/FA-PEG (DOX concentration of 0.1093, 0.2187, 04375, 0.875, 1.75, and 3.5 μg/mL) for 24 and 48 h, respectively. A total of 20 μL MTT solution (5 mg/mL) were added to each well and incubated for 4 h. The crystals were dissolved by adding DMSO. The optical density value of each well was measured at 490 nm by an iMark plate reader (Bio-Rad, Berkeley, CA, United States). All data were obtained in quadruplicate.

### Intracellular ROS Content Measurement

MCF-7 cells were seeded on 6-well plates at a density of 2 × 10^5^ cells per well. Then the cells were incubated with free DOX and DOX@Fe-PDA/FA-PEG (equivalent DOX concentration of 10 μg/mL) for 8 h at 37°C. Afterward, diluted 2′,7′-dichlorofluorescein diacetate (DCFH-DA; Solarbio, Beijing, China), which is a cell-permeable fluorescent probe, were added. Then, the cells were placed in a 6-well plate at 37°C and incubated for another 30 min. The cells were washed for three times with serum-free medium to remove DCFH-DA completely and finally observed using fluorescence microscope.

### Data Analysis Methodology

All experiments were performed at least three times unless otherwise stated. All experimental data were expressed as mean ± SD and both were treated with SPSS 18.0 (SPSS, Chicago, IL, United States).

## Results and Discussion

### DOX@ Fe-PDA/FA-PEG Synthesis and Characterization

**FIGURE 2:**
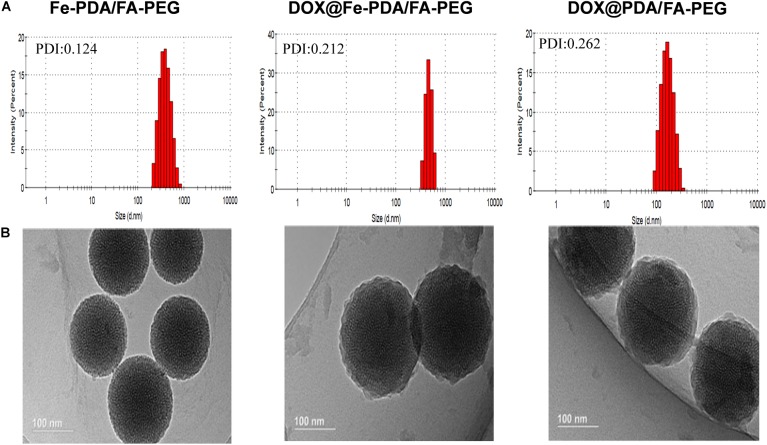
The characterization of different nanoparticl nanoparticles. **(A)** Size distributions of Fe-PDA/FA-PEG, DOX@Fe-PDA/FA-PEG, DOX@PDA/FA-PEG. **(B)** TEM image of PDA/FA-PEG, DOX@Fe-PDA/FA-PEG, DOX@PDA/FA-PEG. Scale bar: 100 nm.

The design and synthetic strategy of DOX@Fe-PDA/FA-PEG is shown in Figure 1. First, the Fe-PDA was synthesized using an oxidative self-polymerization method according to previously literature (Li et al., 2016). In addition, folic acid conjugated PEG was introduced to modify the PDA in enhancing the targeting effect and improving the stability of the NPs. Finally, DOX was loaded via diffusion in an aqueous media. The mean hydrodynamic sizes of DOX@PDA/FA-PEG, DOX@Fe-PDA/FA-PEG and the unloaded Fe-PDA/FA-PEG were 239.5 ± 28.82, 267.7 ± 34.16, and 283.22 ± 21.6 nm, respectively, with a narrow size distribution as demonstrated in Figure 2A. This particle size is theoretically suitable for cellular uptake and tumor cell permeation duo to EPR effect (Maeda, 2015). Zeta potential plays a key role in the stability and penetration through cell membranes for Nps (Bhattacharjee, 2016). Considering the presence of the carboxyl group of FA, the zeta potentials of all NPs are negative (Supplementary Figure 1), thereby indicating that these Nps were stable *in vivo* by electrostatic repulsion, which is the basis of drug delivery (Wu et al., 2011). The zeta potential of Fe-PDA/FA-PEG (-30 mV) is slightly lower than that of Fe-PDA/FA-PEG loaded with DOX (-27.2 mV) (Supplementary Figure 1), thereby suggesting that the positively charged amino groups on DOX partially neutralized the negative charge.

**FIGURE 3:**
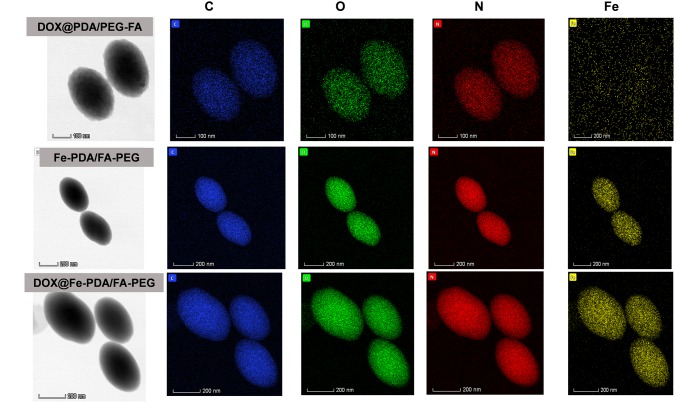
Dark-field image, and corresponding area-elemental mappings of PDA/FA-PEG, DOX@Fe-PDA/FA-PEG, DOX@PDA/FA-PEG. Scale bar: 50 nm.

The morphologies of Fe-PDA/FA-PEG (wihout DOX loaded), DOX@PDA/FA-PEG (without Fe chelated), DOX@Fe-PDA/FA-PEG were observed by TEM. The results revealed that the DOX-loaded PDA/FA-PEG exhibited a spherical and uniform morphology (Figure 2B). The particle size observed by TEM was substantially the same as the particle size measured by DLS. Scanning electron microscopy used to perform accurate elemental analysis of Nps. Using dark field image (DFI) characterization, electron energy loss spectroscopy (EELS), energy dispersive spectroscopy (EDS), and corresponding element mapping (EDS mapping) (Figure 3) clearly show the morphological structure of the nanoparticles and distribution of four elements (C, N, O, Fe). The results showed that the coexistence of C, N, O, and Fe signals coexisted in the EDS spectra of Fe-PDA and Fe-PDA/FA-PEG. The uniform distribution of C, N, O, and Fe was confirmed by EDS element mapping. This result indicated the success and dispersion loads of Fe, PDA, and PEG in the DOX-loaded Fe-PDA/FA-PEG and unloaded Fe-PDA/FA-PEG. However, in the EDS element mapping of PDA, only C, N, and O signals coexisted and were distributed, thereby indicating the success and dispersion load of PDA and PEG in the DOX-loaded PDA/FA-PEG. Further, the FR-IR was performed to evaluat the surface characterization. As shown in Supplementary Figure 2, the, the characteristic peaks of N-H bending vibration appearing at 1512, 1589, and 3250 cm^-1^. The peaks at 1493 and 1445 cm^-1^ can be ascribed to the existence of FA. Compared with PDA, the peaks of PEG at 1128 cm^-1^ (C-O-C stretching) were observed.

### *In vitro* pH-Stimuli Release Study

**FIGURE 4:**
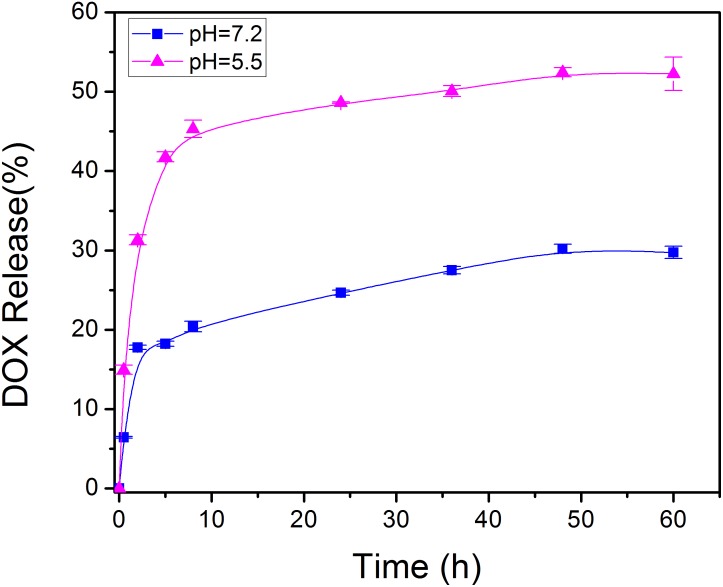
*In vitro* drug release profile of DOX@Fe-PDA/FA-PEG in media with different pH value (pH 7.2 and 5.5).

At a drug to Fe-PDA/FA-PEG feeding ratio of 1:5 in weight, the encapsulation efficiency of DOX in the Fe-PDA/FA-PEG was 76.6 ± 5.2% determined by UV-Vis absorption spectrophotometer. As PDA NPs exist abundant aromatic rings and phenolic hydroxyl groups, the DOX was loaded through π–π stacking and hydrogen-bonding interaction. Subsequently, the pH dependent release capability of DOX@Fe-PDA/FA-PEG was investigated at 37°C under the pH levels of 7.2 and 5.5. The accumulative drug release kinetics curves are shown in Figure 4. The drug release of both the DOX-loaded Fe-PDA/FA-PEG was significantly pH-dependent. As shown in Figure 4, the release of the drug was as low as 25.5% at the of pH 7.2 within 36 h, and even 30.1% within 48 h. However, under acidic conditions, the release amount reached 34.6% within 8 h at the pH of 5.5, and the release rate at 48 h was 47.2%. This indicated that the drug-loaded Nps can cause the drug release under acidic condition, mainly due to the extremely high pH responsiveness of the PDA-modified NPs. This phenomenon allowed the rapid drug release at low pH. Considering the acidic microenvironment of the tumor and intracellular acidic endosomes and lysosomes, drugs are released only after being phagocytized by lysosomes in tumor cells, thereby effectively reducing drug waste and enhancing the antitumor effects by rapidly increasing the lysosome concentration (Duo et al., 2017).

### Cellular Uptake

**FIGURE 5:**
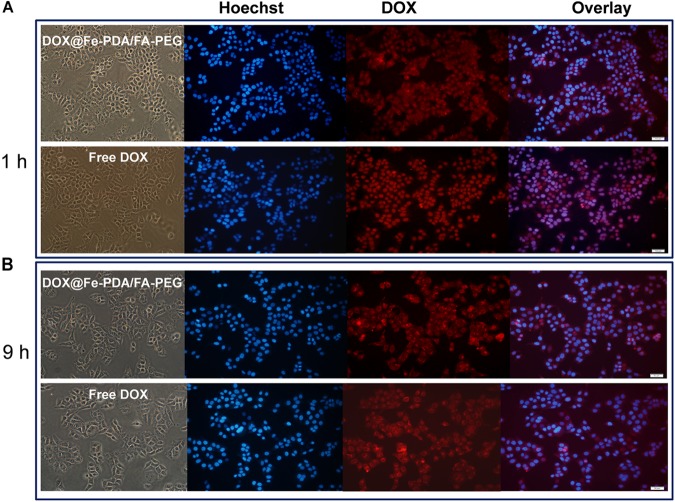
Confocal laser scanning microscopy (CLSM) images of MCF-7 cells after incubation with free DOX, DOX-@ Fe-PDA/FA-PEG for 1 h **(A)**, and 9 h **(B)**. The cells were stained by Hoechst (blue) and drug DOX was red.

To study the cellular uptake and the intracellular distribution, we investigated the intracellular delivery of free DOX by using a confocal microscopy. Figure 5A shows the fluorescence of DOX distrubuted in the cytoplasm and cell nuclei after incubation with free DOX for 1 h. However the red flourescence with NPS observed in nucleus was not obvious. Based on the different intracellular fates of DOX, it was indicated that the NPs were internalized into cell mainly via endocytic pathway. And then we continued to incubate for another 9 h and observed under a fluorescence microscope as shown in Figure 5B. Apparently, the uptake intensities of DOX-loaded NPs was higher than that of free DOX, and it was contributed by the targeting effect of folate receptor. Moreover, the cell uptake intensities of DOX-loaded NPs were positive correlation with incubation time. While the fluorescence intensity of free DOX in the cells is weaker than that of the doxorubicin-loaded NPs, indicating that the intracellular free DOX decays with time. According to the *in vitro* drug release profiles, this phenomenon proves that the DOX-loaded Nps have a sustained release effect, which may help to enhance the cytotoxicity of DOX.

### Cytotoxicity of DOX-Loaded NPs

**FIGURE 6:**
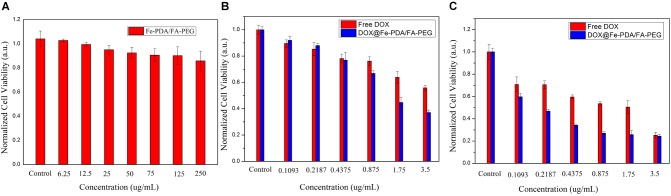
Relative viabilities of MCF-7 cells after incubated with PDA/FA-PEG for 48 h **(A)** and free DOX and DOX@Fe-PDA/FA-PEG at different concentrations 24 h **(B)** and 48 h **(C)**.

To assess the cytotoxicity of DOX@Fe-PDA/FA-PEG, we performed the MTT assays. In order to confirm the high biocompatibility and safety of the NPs, we incubated the Fe-PDA/FA-PEG NPs with MCF-7 cells. As shown in Figure 6A, the Fe-PDA/FA-PEG NPs without drug-loading exhibited a negligible cytotoxicity the concentration ranging from 0 to 250 μg/mL for 48 h. This result suggested that the prepared material possessed high biocompatibility and low cell cytotoxicity. Then, we compared the results of cytotoxicity of free DOX and DOX-loaded Nps at 24 and 48 h. Figure 6B,C shows the cytotoxicity of DOX on MCF-7 was time and dose-dependent. As the DOX concentration and incubation time prolonged, the greater the toxicity of the drug to MCF-7 cell. Apparently, the cytotoxicity of DOX-loaded Fe-PDA/FA-PEG NPs was greater than that free DOX, thereby demonstrating that Fe enhanced the killing effect of DOX on the MCF-7 cells. And it was found that the 48 h of incubation exhibited a considerable killing effect on MCF-7 cells than 24 h. This result further confirmed the sustained release of NPs.

### ROS Detection

**FIGURE 7:**
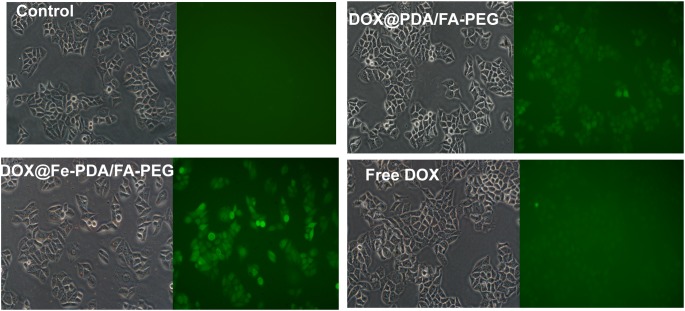
The intracellular ROS stained with DCFH-DA in MCF-7 cells after incubation with free DOX, DOX@Fe-PDA/FA-PEG, DOX@PDA/FA-PEG for 8 h were measured by fluorescence microscopic.

Reactive oxygen species -induced cell death has been a widely uesed strategy for tumor therapy (Matés and Sánchez-Jiménez, 2000; Dixon and Stockwell, 2013; Schumacker Paul, 2015; Zhou et al., 2016). As we know, DOX could activate nicotinamide adenine dinucleotide phosphate oxidases, and further produce ROS, which contribute to anticancer drug-induced toxicity (Chakravarti et al., 2016; Seo et al., 2017). Recently, synergistic approaches by using ROS-producing agents with DOX have attracted considerable attention (Xia et al., 2017). Intriguingly, the presence of Fe (II and III) contributes to the enhanced chemotherapy efficacy by converting the accumulated H_2_O_2_ to the hydroxyl radical via Fenton reactions (Dixon and Stockwell, 2013). To explore the underlying mechanism of enhanced antiproliferating effects of DOX@Fe-PDA/FA-PEG further, we quantified the intracellular ROS by using 2′-7′-dichlorofuorescin diacetate. Compared with the control group, green fluorescence was observed after incubation with DOX and DOX@Fe-PDA/FA-PEG (Figure 7). In addition, cells treated with DOX-loaded Fe-PDA/FA-PEG had the highest fluorescence intensity, thereby indicating the highest ROS production. The results showed that the cells treated with DOX loaded Fe-PDA/FA-PEG can synergistically produce ROS to kill tumor cells. DOX used to undergo redox cycles to generate high H_2_O_2_ levels inside the cancer cells. After endocytosis by tumor cells, the DOX@Fe-PDA/FA-PEG was decomposed by the acidic microenvironment. The elevated H_2_O_2_ of DOX can be further catalyzed by Fe ions via Fenton reaction to generate abundant highly toxic resulting in enhancing anticancer effects of DOX through oxidative damage to DNA, protein, and lipid (Matés and Sánchez-Jiménez, 2000; Schumacker Paul, 2015; Zhou et al., 2016). Previous investigations have developed iron-based nanomaterials, including iron nanometallic glasses and iron oxide, have been employed to upregulation of ROS by using the situ Fenton reaction (Zhang et al., 2016; Liu et al., 2018; Tang et al., 2018). However, current iron-based nanomaterials is far from satisfactory. Some of the nanomaterials such as Fe^0^ nanoparticles (Zhang et al., 2016) and iron oxide nanoplatform (Liu et al., 2018), are difficult to fabricate and the synthetic conditions generally are harsh and complicated. In this work, we synthesized the iron-chelated PDA NPs via a one-pot reaction and the FA-PEG as the surface ligand for tumor homing with a low cost and biocompable biocompatibility. And the pH-stimuli release profiles included being highly selective and logical, and amenable to activation by endogenous stimuli. This strategy present an approach for synergistic combination of ROS and chemotherapy to enhance the anticancer efficacy.

## Conclusion

In this study, we successfully fabricated a novel nanocarrier on the basis of Fe-chelated PDA nanoparticles used for Fe and DOX combined cancer theranostics through ROS over-generation. The obtained DOX@Fe-PDA/FA-PEG Nps had a hydrodynamic size of about 250 nm, and the structure was characterized by DLS, TEM, EDS, and FT-IR. The *in vitro* drug release profile triggered by low intracellular pH indicated that the system demonstrated controlled therapeutic activity. Further, *in vitro* cell uptake studies indicate that DOX-loaded Fe-PDA /FA-PEG can be internalized by MCF-7 cells and exhibited high targeting efficiency due to specific recognition. The *in vitro* experiments demonstrated that DOX@Fe-PDA/FA-PEG trigged the intracellular ROS overproduction, thereby enhancing the therapeutic effect on breast cancer. Taken together, this study provides a strategy to harness Fe-PAD nanocarrier for Fe and DOX combined cancer theranostics.

## Author Contributions

X-JL and W-TL performed the experiments and drafted the manuscript. Z-H-RL and L-PZ prepared and characterized the NPs. C-CG and performed the statistical design of the experiments. W-FZ and D-JD conceived the initial idea. All authors helped to correct and polish the manuscript and read and approved the final manuscript.

## Conflict of Interest Statement

The authors declare that the research was conducted in the absence of any commercial or financial relationships that could be construed as a potential conflict of interest.

## References

[B1] Bhattacharjee S. (2016). DLS and zeta potential – what they are and what they are not? *J. Controll. Release* 235 337–351. 10.1016/j.jconrel.2016.06.017 27297779

[B2] Bray F. Ferlay J. Soerjomataram I. Siegel R. L. Torre L. A. Jemal A. (2018). Global cancer statistics 2018: GLOBOCAN estimates of incidence and mortality worldwide for 36 cancers in 185 countries. *CA Cancer J. Clin.* 68 394–424. 10.3322/caac.21492 30207593

[B3] Camacho K. M. Menegatti S. Vogus D. R. Pusuluri A. Fuchs Z. Jarvis M. (2016). DAFODIL: a novel liposome-encapsulated synergistic combination of doxorubicin and 5FU for low dose chemotherapy. *J. Controll. Release* 229 154–162. 10.1016/j.jconrel.2016.03.027 27034194 PMC5289372

[B4] Chakravarti B. Yang J. Luo Z. Ahlers K. E. (2016). Contribution of NADPH oxidase (Nox)-derived reactive oxygen species (ROS) to doxorubicin-induced cardiomyopathy mediated by regulator of g protein signaling 6 (RGS6). *FASEB J.* 30:939.3 10.1096/fasebj.30.1_supplement.939.3

[B5] Dai Y. Yang Z. Cheng S. Wang Z. Zhang R. Zhu G. (2018). Toxic reactive oxygen species enhanced synergistic combination therapy by self-assembled metal-phenolic network nanoparticles. *Adv. Mat.* 30:1704877. 10.1002/adma.201704877 29315862

[B6] Dayton A. Selvendiran K. Meduru S. Khan M. Kuppusamy M. L. Naidu S. (2011). Amelioration of doxorubicin-induced cardiotoxicity by an anticancer-antioxidant dual-function compound, HO-3867. *J. Pharmacol. Exp. Ther.* 339:350. 10.1124/jpet.111.183681 21799049 PMC3199994

[B7] Dixon S. J. Stockwell B. R. (2013). The role of iron and reactive oxygen species in cell death. *Nat. Chem. Biol.* 10:9. 10.1038/nchembio.1416 24346035

[B8] Duo Y. Li Y. Chen C. Liu B. Wang X. Zeng X. (2017). DOX-loaded pH-sensitive mesoporous silica nanoparticles coated with PDA and PEG induce pro-death autophagy in breast cancer. *RSC Adv.* 7 39641–39650. 10.1039/C7RA05135B

[B9] Fan C. Zheng W. Fu X. Li X. Wong Y.-S. Chen T. (2014). Strategy to enhance the therapeutic effect of doxorubicin in human hepatocellular carcinoma by selenocystine, a synergistic agent that regulates the ROS-mediated signaling. *Oncotarget* 5 2853–2863. 10.18632/oncotarget.1854 24797310 PMC4058050

[B10] Fisher B. Bryant J. Wolmark N. Mamounas E. Brown A. Fisher E. R. (1998). Effect of preoperative chemotherapy on the outcome of women with operable breast cancer. *J. Clin. Oncol.* 16 2672–2685. 10.1200/jco.1998.16.8.2672 9704717

[B11] Fong M. Y. Jin S. Rane M. Singh R. K. Gupta R. Kakar S. S. (2012). Withaferin a synergizes the therapeutic effect of doxorubicin through ROS-mediated autophagy in ovarian cancer. *PLoS One* 7:e42265. 10.1371/journal.pone.0042265 22860102 PMC3408484

[B12] Ge R. Lin M. Li X. Liu S. Wang W. Li S. (2017). Cu2+-loaded polydopamine nanoparticles for magnetic resonance imaging-guided pH- and near-infrared-light-stimulated thermochemotherapy. *ACS Appl. Mat. Interfaces* 9 19706–19716. 10.1021/acsami.7b05583 28553876

[B13] Indermun S. Govender M. Kumar P. Choonara Y. E. Pillay V. (2018). “2 - Stimuli-responsive polymers as smart drug delivery systems: classifications based on carrier type and triggered-release mechanism,” in *Stimuli Responsive Polymeric Nanocarriers for Drug Delivery Applications* Vol. 1 eds Makhlouf A. S. H. Abu-Thabit N. Y. (Sawston: Woodhead Publishing), 43–58. 10.1016/B978-0-08-101997-9.00002-3

[B14] Kemp J. A. Shim M. S. Heo C. Y. Kwon Y. J. (2016). “Combo” nanomedicine: co-delivery of multi-modal therapeutics for efficient, targeted, and safe cancer therapy. *Adv. Drug Deliv. Rev.* 98 3–18. 10.1016/j.addr.2015.10.019 26546465

[B15] Li Y. Xie Y. Wang Z. Zang N. Carniato F. Huang Y. (2016). Structure and function of iron-loaded synthetic melanin. *ACS Nano* 10 10186–10194. 10.1021/acsnano.6b05502 27802021 PMC5295137

[B16] Liu Y. Ai K. Lu L. (2014). Polydopamine and its derivative materials: synthesis and promising applications in energy, environmental, and biomedical fields. *Chem. Rev.* 114 5057–5115. 10.1021/cr400407a 24517847

[B17] Liu Y. Ji X. Tong W. W. L. Askhatova D. Yang T. Cheng H. (2018). Engineering multifunctional RNAi nanomedicine to concurrently target cancer hallmarks for combinatorial therapy. *Angew. Chem. Int. Ed.* 57 1510–1513. 10.1002/anie.201710144 29276823 PMC5898800

[B18] Lynge M. E. Schattling P. Städler B. (2015). Recent developments in poly(dopamine)-based coatings for biomedical applications. *Nanomedicine* 10 2725–2742. 10.2217/nnm.15.89 26377046

[B19] Maeda H. (2015). Toward a full understanding of the EPR effect in primary and metastatic tumors as well as issues related to its heterogeneity. *Adv. Drug Deliv. Rev.* 91 3–6. 10.1016/j.addr.2015.01.002 25579058

[B20] Matés J. M. Sánchez-Jiménez F. M. (2000). Role of reactive oxygen species in apoptosis: implications for cancer therapy. *Int. J. Biochem. Cell Biol.* 32 157–170. 10.1016/S1357-2725(99)00088-6 10687951

[B21] Miao Z.-H. Wang H. Yang H. Li Z.-L. Zhen L. Xu C.-Y. (2015). Intrinsically Mn2+-chelated polydopamine nanoparticles for simultaneous magnetic resonance imaging and photothermal ablation of cancer cells. *ACS Appl. Mat. Interfaces* 7 16946–16952. 10.1021/acsami.5b06265 26196160

[B22] Miller K. D. Siegel R. L. Lin C. C. Mariotto A. B. Kramer J. L. Rowland J. H. (2016). Cancer treatment and survivorship statistics, 2016. *CA Cancer J. Clin.* 66 271–289. 10.3322/caac.21349 27253694

[B23] Russell E. G. Cotter T. G. (2015). “Chapter six - new insight into the role of reactive oxygen species (ROS) in cellular signal-transduction processes,” in *International Review of Cell and Molecular Biology* Vol. 319 ed. Jeon K. W. (Cambridge, MA: Academic Press), 221–254. 10.1016/bs.ircmb.2015.07.004 26404470

[B24] Ryu J. H. Messersmith P. B. Lee H. (2018). Polydopamine surface chemistry: a decade of discovery. *ACS Appl. Mat. Interfaces* 10 7523–7540. 10.1021/acsami.7b19865 29465221 PMC6320233

[B25] Schumacker Paul T. (2015). Reactive oxygen species in cancer: a dance with the devil. *Cancer Cell* 27 156–157. 10.1016/j.ccell.2015.01.007 25670075

[B26] Seo S. U. Kim T. H. Kim D. E. Min K.-J. Kwon T. K. (2017). NOX4-mediated ROS production induces apoptotic cell death via down-regulation of c-FLIP and Mcl-1 expression in combined treatment with thioridazine and curcumin. *Redox Biol.* 13 608–622. 10.1016/j.redox.2017.07.017 28806703 PMC5554966

[B27] Shen Z. Song J. Yung B. C. Zhou Z. Wu A. Chen X. (2018). Cancer therapy: emerging strategies of cancer therapy based on ferroptosis. *Adv. Mat.* 30:1870084 10.1002/adma.201870084 PMC6377162 29356212

[B28] Spiegel D. Y. Koontz B. F. (2018). Meeting the needs of long-term survivors: a testament to success in the care of patients with cancer. *Cancer* 124 2488–2490. 10.1002/cncr.31381 29669188

[B29] Tang Z. Liu Y. He M. Bu W. (2018). Chemodynamic therapy: tumour microenvironment-mediated fenton and fenton-like reactions. *Angew. Chem. Int. Ed.* 58 946–956. 10.1002/anie.201805664 30048028

[B30] Wood R. Mitra D. de Courcy J. Iyer S. (2017). Patient-reported quality of life and treatment satisfaction in patients With HR+/HER2- advanced/metastatic breast cancer. *Clin. Ther.* 39 1719–1728. 10.1016/j.clinthera.2017.07.009 28751098

[B31] Wu H. Liu S. Gong J. Liu J. Zhang Q. Leng X. (2017). VCPA, a novel synthetic derivative of α-tocopheryl succinate, sensitizes human gastric cancer to doxorubicin-induced apoptosis via ROS-dependent mitochondrial dysfunction. *Cancer Lett.* 393 22–32. 10.1016/j.canlet.2017.02.007 28216375

[B32] Wu L. Zhang J. Watanabe W. (2011). Physical and chemical stability of drug nanoparticles. *Adv. Drug Deliv. Rev.* 63 456–469. 10.1016/j.addr.2011.02.001 21315781

[B33] Xi J. Da L. Yang C. Chen R. Gao L. Fan L. (2017). Mn2+-coordinated PDA@DOX/PLGA nanoparticles as a smart theranostic agent for synergistic chemo-photothermal tumor therapy. *Int. J. Nanomed.* 2017 3331–3345. 10.2147/IJN.S132270 28479854 PMC5411169

[B34] Xia J. Inagaki Y. Gao J. Qi F. Song P. Han G. (2017). Combination of cinobufacini and doxorubicin increases apoptosis of hepatocellular carcinoma cells through the Fas- and mitochondria-mediated pathways. *Am. J. Chin. Med.* 45 1537–1556. 10.1142/s0192415x17500835 28946772

[B35] Xu X. Ho W. Zhang X. Bertrand N. Farokhzad O. (2015). Cancer nanomedicine: from targeted delivery to combination therapy. *Trends Mol. Med.* 21 223–232. 10.1016/j.molmed.2015.01.001 25656384 PMC4385479

[B36] Zhang C. Bu W. Ni D. Zhang S. Li Q. Yao Z. (2016). Synthesis of iron nanometallic glasses and their application in cancer therapy by a localized fenton reaction. *Angew. Chem. Int. Ed.* 55 2101–2106. 10.1002/anie.201510031 26836344

[B37] Zhang W. Gai C. Ding D. Wang F. Li W. (2018). Targeted p53 on small-molecules-induced ferroptosis in cancers. *Front. Oncol.* 8:507. 10.3389/fonc.2018.00507 30450337 PMC6224449

[B38] Zheng D.-W. Lei Q. Zhu J.-Y. Fan J.-X. Li C.-X. Li C. (2017). Switching apoptosis to ferroptosis: metal–organic network for high-efficiency anticancer therapy. *Nano Lett.* 17 284–291. 10.1021/acs.nanolett.6b04060 28027643

[B39] Zhou Z. Song J. Nie L. Chen X. (2016). Reactive oxygen species generating systems meeting challenges of photodynamic cancer therapy. *Chem. Soc. Rev.* 45 6597–6626. 10.1039/C6CS00271D 27722328 PMC5118097

