# Supplementary material for: Iron-Chelated Polydopamine Decorated Doxorubicin-Loaded Nanodevices for Reactive Oxygen Species Enhanced Cancer Combination Therapy
Source: Front Pharmacol. 2019 Feb 6;10:75. doi: 10.3389/fphar.2019.00075 (PMC6372743; doi:10.3389/fphar.2019.00075)
Supplement: Supplementary file 1 [file Image_1.pdf]

frontiers

Supplementary Material

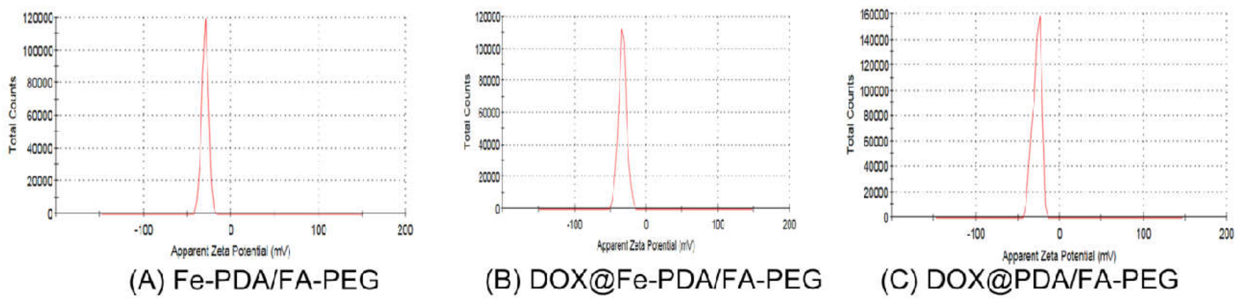

Supplementary Figure 1. Zeta potential measurements of the Fe-PDA/FA-PEG, DOX@Fe-PDA/FA-PEG, DOX@PDA/FA-PEG.

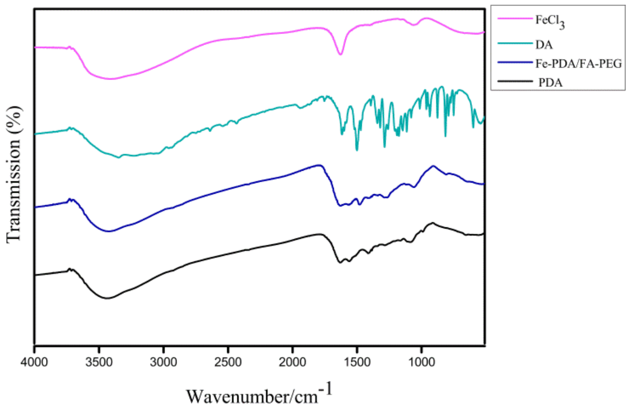

Supplementary Figure 2. FTIR spectra of $FeCl_3$, dopamine (PD), Fe-PDA/FA-PEG, Fe-PDA
